# Supplementary material for: Consistent modelling of material weight loss and gas release due to pyrolysis and conducting benchmark tests of the model—A case for glovebox panel materials such as polymethyl methacrylate
Source: PLoS One. 2021 Jan 28;16(1):e0245303. doi: 10.1371/journal.pone.0245303 (PMC7842909; doi:10.1371/journal.pone.0245303)
Supplement: Attachment — Submitted filename: Response to Reviewers.pdf [file pone.0245303.s001.pdf]

**Academic Editor's comment**	**Author's response**
Please ensure that your manuscript meets PLOS ONEs style requirements, including those for file naming. The PLOS ONE style templates can be found at<br><br>https://journals.plos.org/plosone/s/file?id=wjVg/PLOSOne_formatting_sample_main_body.pdf and<br><br>https://journals.plos.org/plosone/s/file?id=ba62/PLOSOne_formatting_sample_title_authors_affiliations.pdf	We rechecked the PLOS ONE style templates and modified the following two points.<br>i) The format of the captions attached to the figures and tables.<br>ii) If the number of authors of Reference exceeds 6, omit it as "et al." (Ref. 17, 23, 32, 37)

**Reviewer #1's comments**	**Author's responses**
1.Abstract: I think the author should summarize the key important information and results rather than expound the content of the experiment. The abstract should be concise and to the point. And line 43-44, the author did not point out the results of the comparison for the tests on bisphenol-A-polycarbonate.	We reviewed the Abstract description as a whole to concisely state only the key important information and results.<br>In addition, we also revised the abstract to briefly describe the test results for bisphenol A polycarbonate. (Line 53 in Revised Manuscript with Track Changes, Line 42 in Manuscript)
2.In the introduction, it is necessary to add more literature to discuss the previous research of this work in order to further motivate the novelty of this research. Especially for thermogravimetry, the author can refer to the following literature: Journal of Hazardous Materials 402 (2021) 123472; Journal of Cleaner Production 239 (2019) 118113.	We added literature information on previous glovebox fire research. (Lines 66-73 in Revised Manuscript with Track Changes, Lines 54-61 in Manuscript)
3.Line 25, What is the practical significance of this model when evaluating the amount of radioactive material leaking from a nuclear facility during a fire?	We revised the abstract to clarify how the weight loss and the gas release in the model correspond to phenomenon during a fire at nuclear facilities. (Lines 27-28 in Revised Manuscript with Track Changes, Lines 27-28 in Manuscript)

4.Line 43-44, “thermogravimetric (TG)-differential thermal analysis (DTA) (TG-DTA) tests and differential scanning calorimetry tests” should be corrected to “thermogravimetric-differential<br>thermal analysis (TG-DTA) tests and differential scanning calorimetry (DSC) tests”. Similar mistakes were found in Line 115. Please check and revise them.	We checked and revised the abbreviations used in the first appearance. Especially, about TG-DTA, DSC, TG-DTA-MS, and Py-GC-MS. (Lines 80, 81, 133, 139 in Revised Manuscript with Track Changes, Lines 68, 121, 127 in Manuscript)
5.Line 137, The " ~2.5-mg" should be corrected to " ~2.5 mg", Similar mistakes were found through this paper. Please check and revise one by one.	We reviewed the entire the paper and removed unnecessary “-” between numbers and units.
6.Line 192, Could you explain why there are three stages in the pyrolysis of PMMA, and what substances are reacting in there three stages?	We added an explanation for each stage in the pyrolysis of PMMA. (Lines 226-230 in Revised Manuscript with Track Changes, Lines 214-218 in Manuscript)
7.Line 189-197, 203-207, In this section, the pyrolysis behavior of PMMA / PC is lack of in-depth analysis.	Unfortunately, we determined that we do not have enough data to withstand the in-depth analysis. Therefore, we would like to briefly state only the facts obtained as a result of TG measurement here.
8.Line 188, The analysis of kinetic is detailed, but there is a lack of relevant literature support, which can be referred to: Journal of Cleaner Production 253 (2020) 119950.	We checked the recommended literature, but we decided that it would be difficult to use it as a support literature because the analysis method is different from this research. Instead, we added literature information on measurement examples of activation energy in the pyrolysis of PMMA and compared with the results in this study to support the analysis of kinetic. （Lines 279-282 in Revised Manuscript with Track Changes, Lines 264-267 in Manuscript)）<br>In addition, the recommended literature has been cited in the text as an example showing the effectiveness of kinetic analysis for TG data. (Lines 135-136 in Revised Manuscript

	with Track Changes, Lines 122-124 in Manuscript)
9.Conclusion: This part was too long. And the specific results discussion should appear in the "Results and Discussion" section and should not appear in the conclusion.	We revised the conclusion part to be more concise.

**Reviewer #2's comments**	**Author's responses**
• Lines: 78-80 - If the glove box seal is weakened and air in the process room flows into the glove box, the accumulated pyrolysis gas could explode and burn.<br>Reviewer: Could you specify which type of material as normally used as seal?	We added a statement that glove box seals are usually secured by rubber-based materials. (Line 98 in Revised Manuscript with Track Changes, Line 86 in Manuscript)<br>In addition, we clarified the position of this research conducted on PMMA and PC instead of rubber-based materials. (Lines 128-131 in Revised Manuscript with Track Changes, Lines 116-119 in Manuscript)
• Lines: 104-107 - The material used in this study was polymethyl methacrylate (PMMA). PMMA is a flammable material used for glovebox panels. This material was set as the principal target in this study because it occupies most of the mass of combustibles present in the glove box.<br>Reviewer: Is there any other reference in the literature reporting the study of the containment function of the glovebox based on PMMA polymer with a different configuration for the system?	We couldn't find any literature that focused on PMMA and discussed the glovebox containment function except for our previously reported literatures.
• Lines: 112-116 - This weight loss can be regarded as the total amount of pyrolysis gas released.<br>Next, the breakdown of the total released amount for each gas component was investigated by conducting pyrolysis gas quantitative tests. The tests include analysis by	We added reference information containing similar measurements with this study to demonstrate the effectiveness of Py-GC-MS and tube furnace tests. (Lines 140-143 in Revised Manuscript with Track Changes, Lines 128-130 in Manuscript)

pyrolysis (Py)-gas chromatography (GC)-MS (Py-GC-MS) and experiments using a tubular furnace.<br>Reviewer: Similar measurements and experiments were recently published in some references (Primpke, Sebastian, et al. "Comparison of pyrolysis gas chromatography/mass spectrometry and hyperspectral FTIR imaging spectroscopy for the analysis of microplastics." Analytical and Bioanalytical Chemistry (2020): 1-16; Steinmetz, Zacharias, et al. "A simple method for the selective quantification of polyethylene, polypropylene, and polystyrene plastic debris in soil by pyrolysis-gas chromatography/mass spectrometry." Journal of Analytical and Applied Pyrolysis (2020): 104803; Yamane, Shogo, et al. "A data mining method from pyrolyzed products: Pyrolysis-gas chromatography-photoionization-high resolution time-of-flight mass spectrometry and kendrick mass defect analysis for polymer semiconductor poly (3-hexylthiophene)." Journal of Analytical and Applied Pyrolysis 151 (2020): 104923; Bifulco, Aurelio, et al. "Fire and mechanical properties of DGEBA-based epoxy resin cured with a cycloaliphatic hardener: Combined action of silica, melamine and DOPO-derivative." Materials & Design (2020): 108862; Bifulco, Aurelio, et al. "Improving flame retardancy of in-situ silica-epoxy nanocomposites cured with aliphatic hardener: Combined effect of DOPO-based flame-retardant and melamine." Composites Part C: Open Access 2 (2020): 100022). The authors should consider reading	

and citing them during their evaluations along the paper and this introduction section.	
• Lines: 139-141 - The heating rate during heating was controlled as an experimental parameter at 5 °C, 10 °C, 15 °C, or 20 °C/min to kinetically analyze the<br>behavior of sample weight loss because of pyrolysis, i.e., TG data, as described below.<br>Reviewer: Could you specify the ramp temperature at which the TGA measurement was performed?	We specified the ramp temperature of TGA data shown in Figs 3, 4, 5.
• Lines: 193-196 - The reduction amount of TG was 2 wt% in Stage 1, 15 wt% in Stage 2, and 80 wt% in Stage 3, and the residue of pyrolysis was 3 wt%. These weight percentages represent the ratio of the initial weight of the sample. The pyrolysis process began at 150 °C and ended at 440 °C.<br>Reviewer: How do the authors explain these values for the weight losses along the pyrolysis process? Can you describe how the system appears at each stage from a chemical point of view?	We added an explanation for each stage in the pyrolysis of PMMA from a chemical point of view. (Lines 226-230 in Revised Manuscript with Track Changes, Lines 214-218 in Manuscript)
• Lines: 245-247 - Fig 5. An example of an Arrhenius plot for the results of TG-DTA-MS analysis—from the slopes and intercepts of the approximate straight lines, and in Table 1 were determined.<br>Reviewer: Final dot for sentence 247 is missing.	We added the final dot and revised the sentence to fit with the format of PLOS ONE. (Lines 285-287 in Revised Manuscript with Track Changes, Lines 269-271 in Manuscript)<br>We also revised similar errors in the captions for all Figures and Tables.
• Lines:<br>281-283 - Bisphenol-A is a substance equivalent to the monomer material for PC. The species of detectives were like those reported in the literature [17–19].<br>Reviewer: Some references could be cited and	We added information of the recommended references to show the importance of Py-GC/MS in the Bisphenol A measurement and detection. (Lines 320-321 in Revised Manuscript with Track Changes, Lines 304-305 in Manuscript)

inserted on sentence 283 for better supporting the importance of Py-GC/MS in the Bisphenol A measurement and detection (Becerra, Valentina, and Jürgen Odermatt. "Detection and quantification of traces of bisphenol A and bisphenol S in paper samples using analytical pyrolysis-GC/MS." Analyst 137.9 (2012): 2250-2259; Navarro-González, Rafael, Patrice Coll, and Roustam Aliev. "Pyrolysis of γ-irradiated bisphenol-A polycarbonate." Polymer Bulletin 48.1 (2002): 43-51; Bifulco, Aurelio, et al. "Fire and mechanical properties of DGEBA-based epoxy resin cured with a cycloaliphatic hardener: Combined action of silica, melamine and DOPO-derivative." Materials & Design (2020): 108862).	
• Lines: 318-319 - For<br>example, the pyrolysis of bisphenol-A occurs at 265 °C or higher [21].<br>Reviewer: I would suggest adding more references on sentence 319.	We added one reference describing the decomposition temperature of bisphenol-A. (Lines 357-358 in Revised Manuscript with Track Changes, Lines 341-342 in Manuscript)
